# Generic strategic profiling of entrepreneurial SMEs – environmentalism as hygiene factor

**DOI:** 10.1007/s11365-022-00809-2

**Published:** 2022-10-29

**Authors:** Marc Dressler

**Affiliations:** University of Ludwigshafen, Ernst-Boehe-Str. 4, Ludwigshafen, 67059 Germany

**Keywords:** Entrepreneurship, Strategic management, Environmentalism, Population ecology, Sustainability, Ecological strategy, Innovation, Generic strategies, Strategic grouping, Wine industry, Success factors

## Abstract

Climate change, extreme weather phenomena, droughts, fires etc. are just few examples of man-induced impact, jeopardizing the future of mankind. Businesses are increasingly held responsible for and try to manage their environmental impact. Environmentalism and lately sustainability (manifesting an equal pursuit of environmental, social, and economic goals) guide strategic orientation. Whereas large corporations anchor environmentalism in their mission statements and strategic positioning, the strategic reflection of sustainability and especially environmentalism in the business models of small enterprises is less researched. Their entrepreneurship builds on exploiting environmental opportunities and is deemed characteristic for small enterprises, but a lower penetration of strategic instruments paired with a predominant opportunistic behaviour seem to characterize SME´s strategic environmentalism. In order to examine the entrepreneurial environmentalism and the strategic value for SMEs an empiric study leaned on population ecology. An online survey with 291 small enterprises explored environmentalism, strategic profiling, and performance impact in an agricultural and entrepreneurial industry. Study results manifest a positive performance impact of sustainability-oriented and thereby ecologic environmentalism. Ecologic environmental consciousness has been identified for all generic strategic groupings but it separates into two distinctive clusters, one with a process and one driven by market focus. Foremost, eco-centric strategic measures were identified as core levers to increase product quality – a promising finding that secures further strategic ecological environmentalism.

## Introduction

Massive caesural changes manifest in New Normal environments (Ahlstrom et al., 2020). The dynamics of change call for entrepreneurial, strategic management, reemphasizing the need and value of environmentalism (Audretsch et al., 2005; Chen & Liu, 2019; Martín-de Castro, 2020; Panarello, 2021; Teece, 2007). Entrepreneurship chips in desired action, strategy contributes with a sound plan, and environmentalism safeguards to consider the dependency on and responsibility for the environment. Still, empirical depth in order to provide orientation for businesses to navigate in the New Normal is welcomed (Ferreira et al., 2017). An empiric study explores further the complementarity of the concepts, their interaction, and insights into the building bricks by examining the small enterprises in the German wine industry.

The external environment builds a cornerstone in strategic management. Coping with changes in the external environment is a fundamental requirement for companies´ sustainable existence. (Annarelli & Nonino, 2016; Bullough & Renko, 2013; Cameron et al., 2004; Goumagias et al., 2016; Gunasekaran et al., 2011; Leavy, 2014; Mallak, 1998; Weiler, 2016). Identifying opportunities in the external environment nurtures innovation and lays the foundation for superior strategies to outperform in intensifying competition (Papadakis et al., 1998; Veliyath & Fitzgerald, 2000). On the other hand, exploitation of the environment causes environmental and subsequently societal problems (Fortun, 2009; Shrivastava, 1995). Strategic entrepreneurship needs to balance opportunity exploitation and environmental impact (Ayala & Manzano, 2014; Bullough & Renko, 2013; Croitoru et al., 2017; Foster & Kaplan, 2011). Reflecting the paramount managerial importance environmental aspects have, the topic nurtured immense research with often contradicting findings in regards to strategic value and performance impact of environmentalism (Chen & Liu, 2019).

The need to preserve the environment in the course of business activities is not a new idea (Esty & Porter, 1998; Fuchs & Mazmanian, 1998; Porter & Van der Linde, 1995; Prakash, 2000). Natural catastrophes, climate change, pandemic, and ending of natural resources with extensive lethal impact – e.g. starvation – renders environmentalism a guiding managerial principle (Banerjee et al., 2003; Hall et al., 2010; Jacobides et al., 2018). Sustainability, defined as a parallel and synchronized pursuit of economic, societal, and environmental goals, hereby has become a dominant strategic paradigm (Shepherd & Patzelt, 2011) in the hope that sustainable management safeguards the future of our planet. Consumers increasingly consider ecology in their buying decisions pushing companies in the direction of sustainable business models (Kotler, 2011). Indeed, sustainability serves as source to create competitive advantage (Berns et al., 2009). Small and medium enterprises (SME) struggle to redesign their business models in strive for sustainability or ecopreneurship (Johnson & Schaltegger, 2016; Schaltegger & Wagner, 2011). In light of the economic importance of SMEs for societal value creation, their creativity, as well as their relevance in regards to safeguarding the environment, their environmentalism is of paramount importance (European Commission, 2018; Storey, 2003). Still, strategic environmentalism of SME is under-researched.

The study examined market positioning of entrepreneurial small enterprises assessing their strategic concern and responsibility of preserving the natural environment. Population ecology served as underlying theoretical framework, suited to determine successful strategies as mediators of organization and its environment. Overcoming the deficiencies of traditional research on strategy limited to a single firm assessment the group perspective of organizations competing in a similar environment allows to investigate comprehensively and the dynamics of strategy. (Thommen & Boeker, 1986) Herefore, a construct of generic strategic grouping, sustainability measures and therein the ecological initiatives served an empiric analysis of SMEs in one industry and one country. The results contribute to a lack of knowledge on the value of ecological anchored in strategic profiling of small sized enterprises (SME) and in strategic entrepreneurship. Indeed, strategic grouping in the context of entrepreneurship and SMEs (Leitner & Güldenberg, 2010), environmentalism as constituent basis of strategic positioning for SMEs (Perez-Sanchez et al., 2003), and the virtue and value of strategic environmentalism for enterprises that by nature produce natural products (i.e. agriculture and herein wine) is undersearched. The survey assessed generic strategies and the communicated environmentalism of small and medium sized entrepreneurial wineries.

Entrepreneurship declares performance to be a result of seizing opportunities (Dyer et al., 2008; Man et al., 2008). Key characteristics are innovativeness and sustainability (Drucker, 2014). Small business and especially family owned businesses need to be entrepreneurial in order to successfully compete and not to be driven out of the market (Dibrell et al., 2009; Groote & Schell, 2018; Leyer et al., 2018). On the other hand, SME´s entrepreneurial attitude shows in a lower adoption of strategic instruments and more opportunistic implementation of environment-oriented measures (Perez-Sanchez et al., 2003; Deimel, 2008; Frost, 2003; Gibb & Mike, 1985; Leyer et al., 2018). The hereby observed industry of German wine producers is characterized by small, entrepreneurial entities, being dominated by entities of less than 50 employees and domincance of family ownership. Indeed, the searched entities claim high innovativeness with 16% of pioneers and an additional 40% early adopters.

The looked at wine industry brings forward a natural product and thereby production directly impacts nature (Atkin et al., 2011) but also depends on nature and climate (Benson-Rea et al., 2011; Forbes et al., 2013; Schimmenti et al., 2016). Protagonists are exposed to caesural external environment (e.g. liberalization, Covid-19) (Bogonos et al., 2016), and certainly climate change (Malheiro et al., 2010). The German wine industry is characterized by small and entrepreneurial businesses and intensive rivalry (Dressler, 2018a; Loose & Pabst, 2018). Thereby, empiric insights on strategic positioning and the role and impact of environmentalism of the SME is of scientific and practical relevance (Bartunek et al., 2006; Kilduff, 2006; Suddaby, 2006). Exploring wineries´ environmentalism and its strategic business model footprint sheds light on different paths and nuances to environmentalism with strategic, managerial, organizational, and consumer-related implications (Santini et al., 2013; Shrivastava, 1995). The study ambitioned to assess the integration of environmentalism into strategic ambition leaning on resource-based and market-based paradigms. SME are required to consider resource-based limitations and manage valuable and unique resources (Barney, 1991; Peteraf, 1993; Peters et al., 2011). Hart (1995) explicitly expanded the resource-based view by environmental practices to found a natural resource-based view of strategy. Especially for small enterprises with limited leveraging capabilities and funding, environmental adaptation needs reflection in light of resource constraints (Brush et al., 2001; Dressler, 2013). In addition, the market-based perspective to environmentalism provides guidance in light of the growing importance of ethical and green consumerism (Budeanu, 2007; Chatzidakis et al., 2012; Devinney et al., 2010; Germov & Freij, 2010).

The following literature review discloses the richness and breadth of publications on environmentalism in a strategic context, taking into account entrepreneurship and SME. It highlights that environmentalism plays an important role in an agricultural industry context such as the wine business. The apparent scarcity of empiric research examining the relevance and eventual impact of environmentalism on small entrepreneurial businesses motivated the study approach. The methodology section informs about the research questions, the research concept, variables, and the approach. The results section presents the statistical analyses on strategic grouping, sustainability with a focus on environmental measures, and performance impact with a discussion of the findings. A section on limitations and practical implications contains ideas for future research. Last section offers conclusions.

## Literature review

Environmental concern is a key in the theories of environmentalism and ecology. Pepper explicates the epistemic evolution of environmentalism offering a useful summarizing definition of environmentalism as ideology and practices concerned with the environment. (Pepper, 2019) Environmentalism therefore constitues actions in favour of ecological aspects. (Argyrou, 2005; Grizzle, 1994) The interrelationship of human action and natural balance „… is the reason why it is not possible to mark a clear boundary between ecology and environmentalism, ecologists and environmentalists above all in a moment when scientific knowledge spread rapidly and society awakes to its double role: subject and, at the same time, object of study “ (Certomà, 2006) In the following, environmentalism and ecology are therefore used synonymously in their commonality for ecologically oriented behavior. In addition, the concept of sustainability, building on a three-dimensional balanced scorecard to manage enterprises to enable a synchronized pursuit of ecologic, economic and social goals, served to operationalize strategic targets (Torgerson, 1995; Welford, 2013; Paehlke, 2005; Brezuleanu et al., 2015; Butler et al., 1997; Kaplan & Norton, 1998; León‐Soriano et al., 2010) The concept of sustainability not only serves to orchestrate but also to reprioritize the eventually conflicting goals. A transition from shareholder value centrism to stakeholder orientation guided by sustainability is expected to minimize the destruction of natural resources, fragile ecosystems, and counteract climate change (Chan et al., 2016; Nicolaides, 2017; Sundaram & Inkpen, 2004).

### Entrepreneurship, strategic management, and the relevance of the external environmental

Entrepreneurship is vital for economies and societies (Audretsch et al., 2005; Cuervo et al., 2007; Dimitratos et al., 2014; Hermans et al., 2015; Man et al., 2008; Stevenson & Jarillo, 2007; Storey, 2003; Tapsell & Woods, 2010). Entrepreneurship research initially based on personal traits (Dew et al., 2008; Lindow, 2013) with a common definition of entrepreneurs being proactive in decision-making, having a risk taking mentality, paired with creativeness and innovativeness (Covin & Slevin, 1991; Dana et al., 2016; Gartner, 1990). Subsequent research identified further entrepreneurial characteristics (Dimitratos et al., 2014; George & Marino, 2011; Omorede et al., 2015; Robles & Zárraga-Rodríguez, 2015) and expanded the concept from personality-centrism to an organizational perspective (Antoncic & Hisrich, 2003; Amo, 2010; Dimitratos et al., 2014; Stopford & Baden‐Fuller, 1994; Stevenson & Jarillo, 2007). In common, entrepreneurs is defined as the ability to successfully cope with environmental change and to exploit opportunities thereof (Audretsch et al., 2005; Dew et al., 2008; Dyer et al., 2008; Matsuno et al., 2002; Piispanen et al., 2017; Robles & Zárraga-Rodríguez, 2015; York & Venkataraman, 2010).

Entrepreneurship research explains entrepreneurial success to be a result of seizing opportunities in the external environment (Dyer et al., 2008; Man et al., 2008; York & Venkataraman, 2010). Accordingly, the external environment represents a core theme in strategic management when designing successful strategies (Papadakis et al., 1998; Ward & Rebecca, 2000). Strategic management draws from an analysis of the environment, trends, and changes (Bowman & Helfat, 2001; Miller, 1986). Strategy development thus starts by an evaluation of the internal and external environment (Miller, 1987; Papadakis et al., 1998). A positive interpretation of the environment and flexible responses to dynamic change support entrepreneurial behavior, called “bricolage” (Mallak, 1998; Servantie & Rispal, 2018; Zahra & George, 2002). Research on entrepreneurship therefore also ties the concept to family ownership and to SME (Coda et al., 2018; Gomez-Mejia et al., 2011; Lindow, 2013; Man et al., 2008; Olusegun, 2012; Schell et al., 2018; Servantie & Rispal, 2018). The concept of effectuation, where entrepreneurship symptomizes as a clever allocation of available means, illustrates an assumed bias to rely on guts feeling instead of causation-driven planning (Condor, 2020; De Wolf & Schoorlemmer, 2007; Dias et al., 2019; McElwee, 2006; Sarasvathy, 2001; Seuneke et al., 2013). Notably, in the last decade the named theory has attracted a considerable amount of attention in research (Dew et al., 2008; Read & Dolmans, 2012) characterized by a bias that entrepreneurs are environmentally concerned (Berrone et al., 2010). Despite an acknowledged relevance of environmentalism, there is obviously a lack of strategic implementation for small businesses and in the context of generic strategic positioning (Banerjee et al., 2003; Hörisch et al., 2015; Johnson, 2015). In the light of dynamic changes and intensifying rivalry it is of considerable importance to further explore the nature of entrepreneurial decision-making (Smolka et al., 2018).

### Strategic environmentalism: from resilience to ecopreneurship

In regards to strategic environmentalism, the concept of resilience (being able to cope with disastrous external impact) evolved though representing a rather young research stream (Abdullah et al., 2013; Burnard & Bhamra, 2011). Stemming from psychology and medicine, it quickly emphasised a managerial theory with an environmental perspective in order to provide insights how companies can deal with traumatizing and destructive forces (Folke, 2006). These thoughts founded the concept of ecological systems (Goumagias et al., 2016; Holling, 1973). In such context, the exogenous environment not only serves as source for strategic and organizational adaptation but also fosters innovation and sustainability (Folke, 2006; Holling, 1973). The herein postulated need to foresight (Baum et al., 2007) redirects to strategic management, with its characteristic analytic approach of the external as well as the internal environment (Yinan et al., 2011) as core elements (Miller, 1987). Following, organizational alignment has developed as research stream (Lawrence & Lorsch, 1967; Lunenburg, 2012; Miller, 1987; Pertusa-Ortega et al., 2010; Wischnevsky, 2004) where “absorptive capacity” allows dynamic organizations to profit of changes in the environment (Aragón-Correa, 1998; Tian et al., 2018; Zahra & George, 2002). The concepts cumulated in evolutionary and adaptive organizational development (Bueno et al., 2004; Kieser, 1974; Malik & Probst, 1981). Innovation and organizational alignment were identified to be key to cope with turbulent external environment and resulting complexity (Johannessen et al., 1999; D`Aveni, 1994; Denton, 1999; Jenssen & Jørgensen, 2004; Wang & Ahmed, 2004; Hauschildt, 2004; Crossan & Apaydin, 2010; Lloria & Peris-Ortiz, 2014; Tassabehji & Isherwood, 2014).

Business opportunity exploitation might conflict environmental and social interests (Boons & Lüdeke-Freund, 2013; De los Reyes et al., 2017; Porter & Kramer, 2011). The idea of far-reaching, sustainable environmentalism is that companies engage beyond pure compliance to meet social and environmental requirements. Eco-centric business models include ecopreneurship or social entrepreneurship (solving environmental/societal problems), insitutional entrepreneurship (changing regulatory, societal and market institutions) or sustainable entrepreneurship (creating an innovation-based business model that can solve wider market/society problems) (Schaltegger & Wagner, 2011). Research on profit impact and strategic suitability of environmentalism (Albertini, 2013; Porter & Van der Linde, 1995; Aragón-Correa & Sharma, 2003; Melnyk et al., 2003; Shrivastava, 1995) paved ground for sustainability to become a guiding principle (Gladwin et al., 1995; Adner & Zemsky, 2006; Berns et al., 2009; Bonn & Fisher, 2011; Orsato, 2006).

The concept of generic strategic profiling has motivated numerous empiric studies with managerial acclamation practitioners (Dess & Davis, 1984; Porter, 1998; Lüth & Wegener, 2005; Ward et al., 1996; Ward & Rebecca, 2000; Speed, 1989; Campbell-Hunt, 2000; Fiegenbaum & Thomas, 1990; Fiegenbaum & Thomas, 1995; Hitt & Ireland, 1985; Delacroix & Solt, 1987; Santini et al., 2014). Studies on environmentalism within the framework generic strategy are scarce. For the wine industry, Atkin et al. observed that “…a clear business case for environmental management systems exhibited significant differences in cost leadership and differentiation advantages over those without a clear business case for environmental management systems …” (Atkin et al., 2011). Chen and Liu discovered a moderating effect of green innovation and generic profiling (Chen & Liu, 2019).

### Environmentalism in agro-business models

Strategic management in agricultural businesses has received less attention in scientific research (Inderhees, 2007; Seuneke et al., 2013) but recently experiences growing interest (e.g. agricultural entrepreneurship) (Dias et al., 2019). Underdeveloped strategic decision making in the agricultural sector finds explanation in the need for flexible decision making because of the limited predictability of nature (De Wolf & Schoorlemmer, 2007; DeGaetano & Belcher, 2007; Inderhees, 2007; Johnson et al., 2000; Seuneke et al., 2013; Zebisch, 2005). Furthermore, predominantly small companies are stated to lack of capacity or missing capability in strategic management (Leyer et al., 2018; Degravel, 2012; Gibb & Mike, 1985; Deimel, 2008), especially in regards to environmentalism (Aragón-Correa et al., 2008). In addition, the complexity of the external impacts (e.g. climate change) diminishes the applicability of forecasting (Bindi & Howden, 2004; Malheiro et al., 2010). The identified research unanimously expresses the value of strategic decision-making and entrepreneurial traits as well as the need for additional research in the context of environmentalism and agribusiness (Alsos et al., 2011; Condor, 2020; De Wolf & Schoorlemmer, 2007; Dias et al., 2019; McElwee, 2006). This holds especially true for the wine industry (Aytaç et al., 2017; Gilinsky et al., 2014; Haller et al., 2017; Taplin, 2006).

According to Pitelis and Teece (2010), modern firms should think both on the level of innovating for their own sustainable competitive advantage as well as for the sustainability of the industry as a whole, as Benedetto and Corinto (2015) demonstrated by Italian wineries. Agricultural adjustments and initiatives that anticipate possible harmful environmental impacts are of enormous importance for sustainability. Still, operative measures to maintain productivity and quality with potentially negative impact on the environment need adequate research and practical attention (Hannah et al., 2013; Hoemmen et al., 2015). There is a noticeable trend of increasingly using “soft” policy instruments. The institutional context is of relevance when “hard” policy instruments (e.g. laws) with a shift towards environmental governance is intended (Lanoie, 2014). Environmental innovation requires motivation, opportunities and capabilities (Koch & Monßen, 2006) of different actors with the government acting as facilitator (Kooiman, 2003). Hoemmen et al. (2015) reemphasize the value of direct participation, as sustainability initiatives in agriculture only occur if a participatory approach to sustainable development is deployed, whereas a regulatory approach results in a negative impact on economy.

Condor (2020) illustrates the relevance of strategic management and environment in the context of industries dealing with and depending on nature: “… agri-entrepreneurship appears as a new paradigm based on the implementation of deliberate strategies to respond to liberalisation and sustainability.” In the pursuit of synergetic research on strategic management, SME´s, and environmentalism, eco-innovation and sustainability-oriented innovation has been explored (Klewitz & Hansen, 2014). In the agri-food sector, the literature discloses far reaching impact in the form of business model innovation (Tell et al., 2016). Despite existent insights, there is a unanimous request to further explore strategic positioning, environmentalism, and performance impact (Annarelli & Nonino, 2016; Gunasekaran et al., 2011; Hall et al., 2010; Marshall et al., 2005; Mohr, 2016). In regards to strategic profiling of wineries, Atkin et al (2011) concluded a fit of environmental orientation regardless of the chosen strategy.

### German wineries in the context of entrepreneurship and environmentalism

The German wine industry fits an exploration of entrepreneurial environmentalism being highly entrepreneurial and an explicit dependency on nature. German wine producers are predominantly small enterprises with less than 10 full-time employees (BMEL, 2019; Carland et al., 1984; Loose & Pabst, 2018; Olusegun, 2012; Statistisches Bundesamt, 2018). Family ownership dominates the producer landscape (Carland et al., 1984; Dressler, 2018a; Gartner, 1990; Groote & Schell, 2018; Schell et al., 2018). Commonly, wine estates are handed over from one to the next generation. The industry is characterized by intensive rivalry with a squeeze-out of market participants.

Wine estates state climate change as key factor in the external environment. Such an environmental perception, dominated by a topic tied to environmentalism, differs from other industries dominated by rivalry, digitization, and globalization (Agostini & Filippini, 2019; BCG, 2009; Dressler, 2017; Kurth et al., 2019). A positive perception of even excessive environmental threats characterizes protagonists of the German wine industry, supporting entrepreneurship (Dressler, 2018b, 2020). Indeed, wineries´ rich portfolio of creative measures to counter external challenges and profit of opportunities speaks for entrepreneurial bricolage (Dressler, 2020; Servantie & Rispal, 2018).

Wine production is part of agricultural businesses and since vineyards are not safeguarded by greenhouses, nature influences the products, yields, and production processes (Cohen et al., 2009; DeGaetano & Belcher, 2007; Johnson et al., 2000; Malheiro et al., 2010, 2012; Mozel & Thach, 2014; Storchmann, 2012). The term “terroir” expresses the complex relationship of soil, micro-climate, sun, temperature, precipitation and other factors, all of which have an influence on grapes and therefore on wine (Thomas et al., 2013; Van Leeuwen & Seguin, 2006). Hence, natural environment matters when growing and producing wine.

Ecological viticulture extends on about 9% of the German vineyard area (Ahrens, 2020). In a time span of less than 10 years, ecologically treated vineyard surface doubled. Still, ecological planting is far from the political goal of 20% of the vineyard surface in 2030 (Umweltbundesamt, 2020). In order to reach environmental targets of the European Union (manifested in the so called “Green Deal”), individual strategically motivation and resulting measures of the players matter and need to be addressed in order to reach the ambitious goals (Elkerbout et al., 2020; Krämer, 2020; Montanarella & Panagos, 2021; Riccaboni et al., 2021). A range of entrepreneurial environmentalism has been assessed for German wineries with an identification of environmentalism-centered business models (Dressler & Paunović, 2019). Sustainability has gained in relevance in the wine industry, globally (Atkin et al., 2011; Barber, 2010; Benson-Rea et al., 2011; Forbes et al., 2013; Schimmenti et al., 2016) and in Germany (). These insights invite for further exploration of the strategic anchoring of environmentalism in the generic strategies.

## Methodology

Given the high overlap of family ownership, small sized enterprises and entrepreneurship, the chosen population of German wine producers fits to explore environmentalism and entrepreneurship. The surveyed entities are characterized by high entrepreneurship being in a competitive market, where products and marketing require creativity and entrepreneurial ambition. Pioneering spirit of the surveyed entities is very high with more than 50% of the population belonging to pioneers and early adopters. Dependency on the natural environment renders the wineries alert of the value of preserving the nature. On the other hand, agricultural production cannot avoid negative impact on the environment – BCG calculated the negative externalities from agriculture in Germany to exceed 100 million € annually (BCG, 2019) – and consumers expect wine to be a pure natural product. Hence, strategic communication of environmentalism bears the risk to induce a discussion of negative external effects of production, usually not in the interest of the producers.

Entrepreneurship and ecology both are huge research areas with substantial theory and research. In the context of organizational entities and the ambition to integrate the theories ecology of entrepreneurship looks at organizational evolution (Carroll & Khessina, 2005). Leaning on this theoretical approach and the herein positioned cornerstone of agglomeration versus differentiation built a focal point of the hereby reported empiric study. Little research examined strategic decision on environmental profiling in the market in the light of generic strategies (Porter, 1985) and whether to position close or distant to competitors (Deephouse, 1999) in the context of entrepreneurial SMEs (Aragón-Correa et al., 2016; Aragon‐Correa & Leyva‐de la Hiz, 2016; Aragón-Correa et al., 2008; Frost, 2003; Del Brìo & Junquera, 2003; Battisti & Perry, 2011). In order to deliver to this gap an empiric study on strategic positioning with a focus on ecological strategic profiling was realized examining German wineries, all of them SMEs (Menguc & Ozanne, 2005). The study hereby covered not only the terms but the notion of environment (as the external or internal strategic environment), natural environment (input factor or impacted by production), and environmentally oriented activities (i.e. ecological sustainability dimension) from all strategic perspectives. (Menon & Menon, 1997). Assessing an agrarian industry with their essence of natural products is of high interest given the external effects that cannot be avoided. The results thereby feed into the concept of ecological sustainability with its „… dyadic relationships between the organization and entities at the individual, organizational, political-economic, social-cultural, and ecological environment levels “ (Starik & Rands, 1995).

In the endeavour to fill the research gap, two research questions guided the study of environmentalism in generic strategies of small entrepreneurs:

Research question (RQ) 1: What is the performance impact of environmentalism?

Research question (RQ) 2: How does environmentalism determine strategic groupings?

In order to explore the strategic environmentalism, a comprehensive study design approach was chosen (Papadakis et al., 1998). The model tested for the interaction of environmental focus and generic grouping (Chen & Liu, 2019). In comparison to Chen and Liu´s model to assess strategic effects of environmentalism, our study design (1) refrains from an assessment of the rivalry in reflection of a single-industry analysis; (2) five strategic groupings make up for our model therefore exceeding a dichotomous strategic profiling of cost versus differentiation; and (3) our study assesses environmentalism in a two-step analysis (firstly sustainability via its three pillars and subsequently by four measures of environmentalism for each category). The study thereby builds on a model of independent variables to measure strategic grouping, sustainability, environmentalism, and dependent variables to measure performance impact.

Table 1 details the variables used in the questionnaire in addition to descriptive information (e.g. age, size, governance):

**Table 1:** Variables of the applied model

**Variables**	**Specification**	**Scale**
**Strategy**		
**Generic strategies in wine**	cose leader	Most relevant (1 out of 5)
	price-value	
	quality leader	
	premium	
	niche	
**Sustainability variables**		
**Environmental sustainability measures**	eco-friend viticulture	Evaluation of relevance Likert scale: 0 = insignificant 1 = low 2 = average 3 = high 4 = very high
	nature preservation	
	saving resources	
	waste minimization	
**Economical sustainability measures**	sustainability as strategic goal	
	longterm corporate stability	
	profitableness / economic success	
	reliable customer relationship	
**Social sustainability measures**	valuing work environment	
	work-life balance	
	reliable partner relationship	
	philantrophy	
**Performance variables**		
**Quantitative performance variables**	revenue	Performance evaluation Likert scale: 1 = very poor 2 = poor 3 = satisfactory 4 = good 5 = very good
	profit	
	capital structure	
	cost situation	
	market share	
**Qualitative performance variables**	product quality	
	service qualtiy	
	new customers acquisition	
	customer loyality	
	market positioning	
	market development	
	personal satisfaction	

Porters´ two-dimensional strategic grouping by competitive advantage and competitive scope (Porter, 1988), the foremost used framework in science and practice to define strategies (Aerts et al., 2007; Barney, 1997, 2001; Gonzlez-Benito & Suárez-González, 2010; Hutzschenreuter & Kleindienst, 2006; Islami et al., 2020; Pertusa-Ortega et al., 2010), served to cluster the generic strategic groupings. The wineries were asked to opt for their characterizing strategic grouping to be picked out of five strategies in reference (cost leadership, premium differentiation, niche positioning, price-quality or quality leadership). The survey provided additional explanation and abstract examples for the options of strategic groupings. This approach has been tested in three prior panels.

Environmentalism was evaluated by assessing the wineries´ sustainability measures with four key measures in every sustainability category (Neely & Hii, 1998). The variables assessed the relevance of the measures on a 5-point Likert scale. The model, variables, and the scale hence allowed statistical analyses in order to assess significance of relationships (Boone & Boone, 2012; Backhaus et al., 2016, Hair et al., 1998).

In regards to the dependent variables, the study followed literature´s recommendations to use multiple success measures, to cover quantitative as well as qualitative measures, and to rely on subjective perception of the entrepreneurs when examining SME performance (Wacht et al., 2016; Sorich & Rivera, 2018; Saunila, 2014; Simpson et al., 2012; Santini et al., 2014; Maruso & Weinzimmer, 1999). Seven qualitative success measures (e.g. satisfaction, product quality …) (Scott Morton & Podolny, 1999) and five quantitative variables (e.g. revenues, market share etc.) (Deimel, 2008; Santini et al., 2014) constitute the performance evaluation. Hereby, the model reflected that „… entrepreneurs measure success beyond economic returns “ (Wacht et al., 2016). Self-assessments of the entrepreneurs (Maruso & Weinzimmer, 1999) suits SME performance evaluation (Chen & Liu, 2019). Furthermore, the approach covered both, the business´ as well as the entrepreneur´s perspective as proposed in the literature (Sparrow & Cooper, 2014). To rely on personal satisfaction when assessing results (Scott Morton & Podolny, 1999) and self-assessment by the respondents (Santini et al., 2014) reflects SME particularities (Maruso & Weinzimmer, 1999) and is recommended in the wine specific literature. All variables have been tested in the three prior panels on strategy and sustainability of German wineries in a two year sequence starting in 2012. It allowed to refer and validate in the results section at instances with insights from the preceding studies.

From November 2018 until March 2019, more than 2,000 wineries were invited to participate in an online survey assessing the strategic building blocks (Patton, 2005). A pre-test of the questionnaire resulted in minor adaptations. The participants were promised anonymity and received a comprehensive summary of the survey results at request. Anonymity was provided following a two-step process: they accessed a survey webpage and received an individualized access number. Every winery had access to only one code. The survey resulted in n = 291 useable interviews. Survey data were analysed by SPSS statistics 24 software. A variety of analyses was performed acknowledging the scale levels, including regression, boxplots, rank analyses, and ANOVA.

The survey population consisted predominantly of small, family owned enterprises (see Table 2). More than 60% of the participants employ less than five fulltime employees with annual revenues of less than 100,000 Euros. Only 10% employed more than 25 people, but none reached or exceeded 100 employees – the whole population therefore quantified as small or micro enterprises (European Commission, 2018). More than 80% of the participants were family wineries, the owners filled out the survey. Core business of the participants was wine production and sales:

**Table 2:** Population and descriptive data

Sample Characteristics	N	% of total	% within categ. resp.
**Position**	279		
Owner	186	64%	67%
Manager	66	23%	24%
Others	27	9%	10%
**Organization**	286		
Governance by family / owner	240	82%	84%
Governance by manager (independent wineries)	13	4%	5%
Governance by manager (Cooperatives)	33	11%	12%
**Number of Employees**	280		
No employees / family business	59	20%	21%
Less than 5 employees	121	42%	43%
5—24 employees	72	25%	26%
25 and more	28	10%	10%
**Annual production**	254		
Less than 1,000 hectoliters	103	35%	41%
1,000—5,000 hectoliters	107	37%	42%
5,000 hectoliters and more	44	15%	17%
**Annual Sales (revenue)**	222		
Less than 100,000 Euros	14	5%	6%
100,000 < 500,000 Euros	76	26%	34%
500,000 < 2 million Euros	86	30%	39%
2 million Euro and more	46	16%	21%
**Value creation**	288		
Viticulture	246	85%	85%
Vinification	261	90%	91%
Winesales	268	92%	93%
Winetourism	171	59%	59%
**Strategic group**	291		
cost leader	10	3%	3%
price-value	128	44%	44%
quality leader	62	21%	21%
premium	43	15%	15%
niche	48	16%	16%

## Results and discussion

The participants group into mix of generic strategies, dominated by price-value positioning and second in place quality-leadership: 44% of the population picked price-value strategy to be characteristic, 21% realize quality leadership, 16% state niche and 15% premium strategy, and 3% opt to represent a generic cost leadership strategy. The stated distribution across the generic groups reflects industry fragmentation in line with prior panels.

**Fig. 1:**
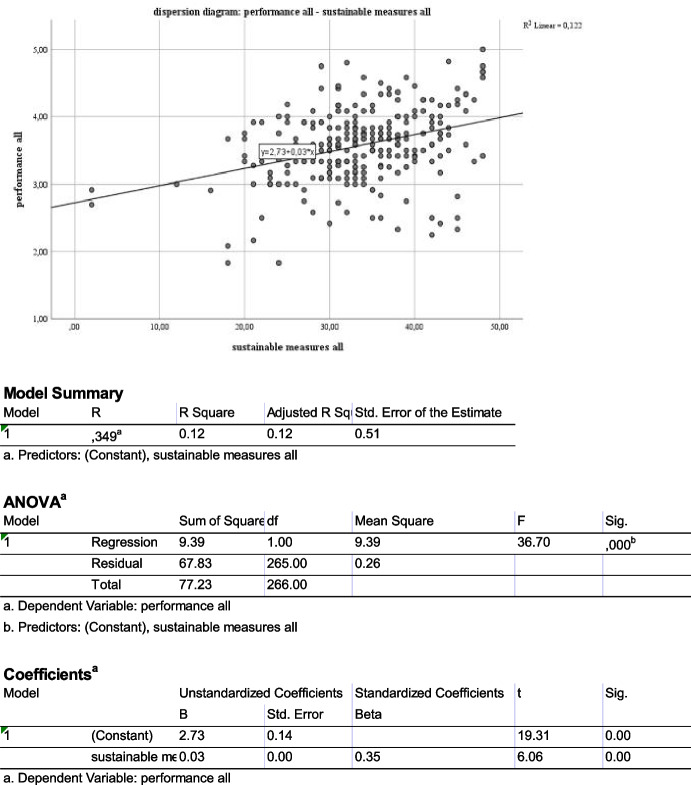
Dispersion diagram & regression of sustainability and performance

In order to examine the relevance and impact of sustainability and respectively environmentalism, the Likert values of the sustainability measures were summed up allowing to evaluate the relevance for the population. The simple dispersion diagram visualizes a positive correlation to the performance variables. Performance was measured as mean Likert value of the five quantitative and seven qualitative performance variables (1 = very poor; 5 = very good) (see Fig. 1).

ANOVA, appropriate for Likert scale based variables (Boone & Boone, 2012), was deployed to analyse the significance of the correlation. The visually observable dependency is significant. Looking at the underlying sustainability dimensions, they all qualify as significant correlation. The economical sustainability turns out to provide the highest R-Squared (coefficient of determination) value (Table 3).

**Table 3:** Model summary of sustainability dimensions on performance variables

Model	R	R Square	Adjusted R Square	Std. Error of the Estimate
**Environmental sustainable measures**				
1	,201^a^	0.041	0.037	0.52589
**Economical sustainable measures**				
1	,366^b^	0.134	0.131	0.49536
**Model Summary**				
1	,298^c^	0.089	0.086	0.50932

^a^Predictors: (Constant), Environmental sustainable measures all

^b^Predictors: (Constant), economic sustainable measures all

^c^Predictors: (Constant), social sustainable measures all

The results illustrate that pioneering in sustainability obviously has a positive performance impact, in spite of cost effects (Melnyk et al., 2003; Huang et al., 2014; Albertini, 2013). These insights elucidate a striking gain in relevance of sustainability within the strategic measures of wineries (Dressler, 2018b). In line with expectations and prior studies, the survey hereby discloses that for wine producers, the ecological environment is of high importance (Esty & Porter, 1998; Orsato, 2006; Reinhardt, 1998). In an industry dealing with a natural product, as is the case for the searched wine producers, spanning the boundary with a focus on the external ecological environment manifests as strategic core.

In order to seize the environmental foundation in the strategic groupings, mean values of the stated importance for all assessed sustainability measures allowed statistical analyses. ANOVA results confirm that environmentalism as well as all three dimensions of sustainability significantly impact the strategic grouping (see Table 4) with a high level of significance. Quality leadership outperforms all other strategies. For cost leadership and price-value strategy, economic sustainability is most important, they are laggards in environmentalism. Niche players show a characteristic profile led by social sustainability, followed by environmentalism more important than economic performance. In regards to environmentalism, quality leaders and premium wineries are most engaged.

**Table 4:** ANOVA of environmentalism & sustainability and strategic grouping

**Report**	**Importance of Sustainable Measures (mean)**						
strategy		environmental	economical	social	all		
cost leader	Mean	2.0500	2.2500	2.1250	2.1042		
	N	10	8	8	8		
	Std. Deviation	1.22554	1.06066	0.81284	0.90276		
price-value	Mean	2.5768	2.7927	2.6563	2.6846		
	N	127	123	120	116		
	Std. Deviation	0.74902	0.74843	0.70119	0.57439		
quality leader	Mean	2.8911	3.0533	2.8475	2.9425		
	N	62	61	59	58		
	Std. Deviation	0.76101	0.57212	0.67437	0.52779		
premium	Mean	2.7917	3.0366	2.9302	2.9167		
	N	42	41	43	38		
	Std. Deviation	0.72397	0.55216	0.61559	0.51843		
niche	Mean	2.7500	2.6848	2.8351	2.7595		
	N	47	46	47	44		
	Std. Deviation	0.97802	0.83239	0.75771	0.77159		
Total	Mean	2.6858	2.8522	2.7545	2.7696		
	N	288	279	277	264		
	Std. Deviation	0.81244	0.72893	0.70789	0.62279		
**ANOVA Table**							
			Sum of Squares	Df	Mean Square	F	Sig
environmental * strategy	Between Groups	(Combined)	8.830	4	2.208	3.459	0.009
	Within Groups		180.606	283	0.638		
	Total		189.437	287			
economic * strategy	Between Groups	(Combined)	8.486	4	2.122	4.175	0.003
	Within Groups		139.227	274	0.508		
	Total		147.714	278			
social * strategy	Between Groups	(Combined)	6.472	4	1.618	3.338	0.011
	Within Groups		131.835	272	0.485		
	Total		138.307	276			
all * strategy	Between Groups	(Combined)	6.941	4	1.735	4.727	0.001
	Within Groups		95.069	259	0.367		
	Total		102.010	263			
**Measures of Association**							
	Eta	Eta Squared					
environmental * strategy	0.216	0.047					
economic * strategy	0.240	0.057					
social * strategy	0.216	0.047					
all * strategy	0.261	0.068					

For the analysis of the four variables of ecological environmentalism Kruskal–Wallis was deployed, reflecting scale levels in accordance to Likert items (see Table 5):

**Table 5:** Ranking analysis / Kruskal–Wallis of environmental variables

**NPar Tests**				
**Kruskal–Wallis Test**				
**Ranks**				
strategy		N	Mean Rank	
eco-friendly viticulture	cost leader	10	75.35	
	price-value	128	130.79	
	quality leader	62	165.52	
	premium	43	164.40	
	niche	48	159.59	
	Total	291		
nature preservation	cost leader	10	91.35	
	price-value	127	126.52	
	quality leader	62	162.71	
	premium	43	172.97	
	niche	47	157.39	
	Total	289		
saving resources	cost leader	10	125.65	
	price-value	127	139.80	
	quality leader	62	161.50	
	premium	44	141.09	
	niche	47	148.14	
	Total	290		
waste minimization	cost leader	10	132.00	
	price-value	127	144.18	
	quality leader	62	151.38	
	premium	43	137.12	
	niche	47	148.79	
	Total	289		
**Test Statistics**^a,b^	environmentally-friendly viticulture	nature preservation activities	saving resources	waste minimization
Kruskal–Wallis H	19.401	20.818	3.947	1.257
df	4	4	4	4
Asymp. Sig	0.001	0.000	0.413	0.869

^a^Kruskal Wallis Test

^b^Grouping Variable: Strategie

Within the ecological environmentalism, no statistical significance for the two levers saving resources and waste minimization is observable. To the contrary, the two other strategic ecological levers of eco-friendly viticulture and nature preservation show high significance. Quality leaders and premium providers stand out in taking care of the natural environment. Both score highest for eco-friendly viticulture and nature preservation. A post-hoc test served to validate and to identify the variable interactions (see Table 6):

**Table 6:** Post-hoc test of the two significant environmentalism variables

Sample 1-Sample 2	Test Statistic	Std. Error	Std. Test Statistic	Sig	Adj. Sig.^a^	Cohen's d
**Pairwise Comparisons of strategy for eco-friendly viticulture**						
cost leader-price-value	-55.439	26.520	-2.090	0.037	0.366	
cost leader-niche	-84.244	28.076	-3.001	0.003	0.027	0.317
cost leader-premium	-89.045	28.356	-3.140	0.002	0.017	0.320
cost leader-quality leader	-90.166	27.524	-3.276	0.001	0.011	0.323
price-value-niche	-28.805	13.670	-2.107	0.035	0.351	
price-value-premium	-33.606	14.237	-2.361	0.018	0.182	
price-value-quality leader	-34.727	12.497	-2.779	0.005	0.055	0.312
niche-premium	4.802	16.959	0.283	0.777	1.000	
niche-quality leader	5.922	15.528	0.381	0.703	1.000	
premium-quality leader	1.121	16.029	0.070	0.944	1.000	
**Pairwise Comparisons of strategy for nature preversation**						
cost leader-price-value	-35.174	26.196	-1.343	0.179	1.000	
cost leader-niche	-66.044	27.775	-2.378	0.017	0.174	
cost leader-quality leader	-71.360	27.180	-2.625	0.009	0.087	0.308
cost leader-premium	-81.615	28.001	-2.915	0.004	0.036	0.315
price-value-niche	-30.780	13.617	-2.267	0.023	0.234	
price-value-quality leader	-36.186	12.357	-2.928	0.003	0.034	0.315
price-value-premium	-46.441	14.072	-3.300		0.010	0.324
niche-quality leader	5.316	15.426	0.345		1.000	
niche-premium	15.571	16.831	0.925		1.000	
quality leader-premium	-10.255	15.828	-0.648		1.000	

Each row tests the null hypothesis that the Sample 1 and Sample 2 distributions are the same. Asymptotic significances (2-sided tests) are displayed. The significance level is,05

^a^Significance values have been adjusted by the Bonferroni correction for multiple tests

The null hypothesis, that the distribution of “eco-friendly viticulture” and of “nature preservation” are the same across categories of strategy, could be rejected (independent-Samples Kruskal–Wallis test). Cohen´s d-values identify two distinguished ecological environmental approaches of the groupings cost leadership and price leadership against premium and quality leaders. Ecological viticulture is of highest relevance for the strategies quality leadership and premium. In their ecologically-oriented activities, cost-leaders and price-value clusters are more process-driven (waste reduction / minimizing waste).

The findings hereby provide supportive evidence to Atkin et al. (2011) that a) environmentalism suits all generic strategies and b) environmentalism has a positive impact on the performance. Furthermore, in support to Chen & Liu exploring Chinese enterprises, the findings illustrate that environmentalism constitutes entrepreneurial gist of the matter for differentiation. Besides additional information having explored five strategic groupings, the findings elucidate the strategic lever of environmentalism on product quality (see Table 7):

**Table 7:** Analysis of eco-friendly viticulture on the performance variable product quality

**Pairwise Comparisons of product quality for eco-friendly viticulture**					
Sample 1-Sample 2	Test Statistic	Std. Error	Std. Test Statistic	Sig	Adj. Sig.^a^
very poor-poor	-93.250	87.059	-1.071	0.284	1.000
very poor-good	-129.000	78.126	-1.651	0.099	0.987
very poor-satisfactory	-133.091	79.618	-1.672	0.095	0.946
very poor-very good	-157.686	78.238	-2.015	0.044	0.439
poor-good	-35.750	39.446	-0.906	0.365	1.000
poor-satisfactory	-39.841	42.326	-0.941	0.347	1.000
poor-very good	-64.436	39.669	-1.624	0.104	1.000
good-satisfactory	4.091	17.770	0.230	0.818	1.000
good-very good	-28.686	9.895	-2.899	0.004	0.037
satisfactory-very good	-24.595	18.258	-1.347	0.178	1.000

Each row tests the null hypothesis that the Sample 1 and Sample 2 distributions are the same. Asymptotic significances (2-sided tests) are displayed. The significance level is,05

^a^Significance values have been adjusted by the Bonferroni correction for multiple tests

A correlation with high significance of the most prominent ecological environmental variable and product quality seems key for strategic environmentalism. Finding product quality to be positively determined by environmentalism indicates a paradigmatic shift in the industry. For a long time, the wine industry differed from other food categories. General consumer perception, with the exception of biological wine buyers, was that eco-centric wine estates jeopardize premium wine quality wine (Ipsos, 2015; Janssen & Hamm, 2013). Indeed, many wineries chose not to communicate eco-certification on their labels (Delmas & Grant, 2008). Indeed, premium wineries, although predominantly certified as ecological vintners, often refrained from according active communication. The stated growth in consumer awareness and request for sustainability and visible resource allocation from the producers illustrate a change towards more eco-centrism (Dressler, 2021; Fader, 2012; Mend, 2012). Modern consumer demands regarding agricultural products continue to move the food production towards natural farming and agriculture meeting environmental, ethical, social and health concerns (Forbes, 2009; Nosi & Zanni, 2004). Profiting of the consumer change but considering the observed resource drain support that environmentalism builds upon a market-based and resource-based approach to sustainability. The survey results trigger the notion that in the searched wine industry environmentalism is becoming “strategic must”. It is therefore important for wine producers to start with sustainability evaluation of own resources and business practices before proceeding further to sustainable food markets and sustainable consumers. Generic strategies profiling on premium wines and the quality of the product are expressively required to manage the ecological environment accordingly. Niche players apparently can draw on environmentalism to differentiate. The identified relevance of environmentalism and performance impact underlines the importance of strategic environmentalism and according anchoring in the business models.

The positive impact of environmentalism and entrepreneurship on performance underline that eco-centric entrepreneurship creates value. Despite the very high relevance environmentalism, the analyses did not disclose environmentalism-induced innovation. Obviously, managing the environmental impact consumes management attention and draws on limited resources in an industry that produces a natural product. As a result, the industry shows a strong product focus also in regards to their innovation portfolio (Dressler, 2013). Product decisions require a very long time since vineyards usually are planted for more than 30 years and the first years are without yields.

## Limitations and practical implications

The study´s findings are limited having explored only one industry in just one country. Furthermore, the observed agriculturally engaged population with its dependency on nature and the ecological environment further limit general application of the findings. In consideration of the entrepreneurial structure of the searched industry and the challenges of increasing competition, the derived foundation of environmentalism in the different generic strategies can either motivate comparable research in other SME industries or allow hypothesis generation and validation. Still, the provided evidence on relevance of environmentalism as core lever to increase product quality and its importance in case of differentiation especially in the premium segment arguably reflect a societal change. Exploitation of the planet and devastating impact of mankind shifts environmentalism further to become a must in strategic activities for differentiation.

For industry practitioners, the implications differ by strategic grouping. Environmental and eco-friendly production is a must for premium strategy grouping or ambitioning. Niche strategies can further leverage on environmental profiling. Quality leadership requires additional eco-centric activities. Cost leaders are well advised to communicate their ecological measures in regards to resource savings and waste reduction to gain reputation. Furthermore, the searched industry departs from product-centrism as well as a male domination in the family businesses. This transition is expected to alter the strategic profiling and the insights help to secure resilience and survival in the market restructuring. Indeed, the searched industry profits of environmental profiling and environmentalism-founded innovation to address emerging needs of customers. This enables the players to differentiate and to win customers and market share in an increasingly competitive and squeezing-out industry. Such a reading of the results of this study is of value for politics and associations trying to increase ecological penetration to meet communicated targets: environmentalism is of importance for all players regardless of their strategic grouping, and measures and motivation should not be limited to ecologically certified actors. The ambitious goals, predominantly posed by politics, can only be achieved motivating an industry-wide change. Hereby, the notion of environmentalism-based innovation shows room for improvement.

## Conclusion

Climate change, extreme weather phenomena, droughts, fires etc. are just few examples of man-induced impact, jeopardizing the future of mankind. Businesses are increasingly held responsible for and try to manage their environmental impact. Environmentalism and lately sustainability manifesting an equal pursuit of ecologic, social, and economic goals, increasingly guide strategic orientation. Whereas large corporations position environmentalism in their mission statements with according corporate level departments, the strategic reflection of sustainability and especially environmentalism in the business models of SME is less obvious. Entrepreneurship builds on exploiting environmental opportunities and is deemed characteristic for small enterprises with an expressed need of synchronized environmentalism. The hereby reported study explored environmentalism in strategic groupings of small-businesses in an agricultural industry.

Against posited expectations that small entrepreneurs lack the resources and capabilities for strategic environmentalism, the analyses support high relevance of sustainability and environmentalism in an entrepreneurial industry dealing with a natural product. Indeed, environmentalism was discovered to be anchored across generic strategies, with different focal points and individualized portfolio of measures – hence speaking for entrepreneurial management of sustainability. The survey disclosed a positive impact of environmentalism on quantitative and qualitative performance indicators, justifying and motivating environmental engagement. Indeed, the results manifest a positive reputational effect of environmentalism as product quality increases by environmental measures. Thereby, environmentalism becomes an imperative for strategies. Furthermore, the identified environmentalism-based strategic groupings show an ecological environmental agglomeration of the generics strategies of cost leadership and price leadership against premium and quality leaders. Whereas the first grouping strongly profits of process-oriented cost implications draws the second grouping advantages for their differentiation strategies. Environmentally oriented entrepreneurial business models obviously create value.

For the searched wine industry, environmentalism is transforming into a strategic “hygiene factors” rather than a satisfier. Environmentalism is therefore to be implemented with different measures in all generic strategies. The insights call for industry-wide motivation for environmentalism in order to meet the communicated political ambitions of increasing ecological vineyard surface instead of turning few players into ecopreneurs. Still, the searched industry might be able to profit of more profiling on environmentalism-founded innovation to address emerging needs of customers. This enables the players to further differentiate and to win customers and market share in an increasingly competitive and squeezing-out industry.
